# Zebrafish in the sea of mineral (iron, zinc, and copper) metabolism

**DOI:** 10.3389/fphar.2014.00033

**Published:** 2014-03-06

**Authors:** Lu Zhao, Zhidan Xia, Fudi Wang

**Affiliations:** ^1^Department of Nutrition, Center for Nutrition and Health, School of Public Health, School of Medicine, Zhejiang UniversityHangzhou, China; ^2^Institute of Nutrition and Food Safety, Zhejiang UniversityHangzhou, China

**Keywords:** zebrafish, trace elements, minerals, iron, copper, zinc, metabolism

## Abstract

Iron, copper, zinc, and eight other minerals are classified as essential trace elements because they present in minute *in vivo* quantities and are essential for life. Because either excess or insufficient levels of trace elements can be detrimental to life (causing human diseases such as iron-deficiency anemia, hemochromatosis, Menkes syndrome and Wilson's disease), the endogenous levels of trace minerals must be tightly regulated. Many studies have demonstrated the existence of systems that maintain trace element homeostasis, and these systems are highly conserved in multiple species ranging from yeast to mice. As a model for studying trace mineral metabolism, the zebrafish is indispensable to researchers. Several large-scale mutagenesis screens have been performed in zebrafish, and these screens led to the identification of a series of metal transporters and the generation of several mutagenesis lines, providing an in-depth functional analysis at the system level. Moreover, because of their developmental advantages, zebrafish have also been used in mineral metabolism-related chemical screens and toxicology studies. Here, we systematically review the major findings of trace element homeostasis studies using the zebrafish model, with a focus on iron, zinc, copper, selenium, manganese, and iodine. We also provide a homology analysis of trace mineral transporters in fish, mice and humans. Finally, we discuss the evidence that zebrafish is an ideal experimental tool for uncovering novel mechanisms of trace mineral metabolism and for improving approaches to treat mineral imbalance-related diseases.

## Introduction

As children of the Earth, humans are intimately connected to our surroundings in many ways, and the relationship between humans and minerals is perhaps the most enigmatic. Based on their necessity for life and their limited quantities with the human body, 11 elements are classified as trace minerals, including iron (Fe), zinc (Zn), copper (Cu), selenium(Se), manganese(Mn), iodine(I), molybdenum(Mo), fluorine (F), cobalt (Co), chromium (Cr), and vanadium (V) (Fraga, 2005).

Metal trace minerals are biologically active primarily as metalloproteins formed by conjugating or binding with various protein partners. Metalloproteins account for approximately half of all proteins and perform a wide range of biological functions as enzymes, transporters and signal transducers. In metalloproteins, metal trace minerals are essential components, acting at the enzyme's active site or by stabilizing the protein's structure. Trace mineral deficiencies can cause a number of diseases that can be mild, severe, or even fatal. Conversely, excess levels of trace minerals can be toxic. For example, excess iron or copper produces reactive oxygen species (ROS) via the Fenton reaction, resulting in lipid peroxidation, DNA damage, altered calcium homeostasis, and cell death (Stohs and Bagchi, 1995). Excess levels of redox-inactive metals such as zinc are also harmful; the accumulation of zinc triggers neuronal death in the brain and induces copper deficiency. Thus, the balance of endogenous trace minerals must be tightly regulated.

Organisms have evolved comprehensive systems for maintaining trace element homeostasis; these systems are composed primarily of transport proteins, storage proteins, and some hormones. Our current knowledge regarding these regulatory factors has come primarily from studies using model organisms ranging from yeast to mice. Among these species, the zebrafish (*Danio rerio*) has been a valuable vertebrate system with several unique advantages. First, their small size, high fertility rate, and rapid development make zebrafish an ideal model for large-scale genetic screens. Secondly, because they are fertilized *ex vivo* and are optically transparent, zebrafish embryos are ideally suited for experimental techniques such as gene knockout/knockdown and overexpression. Because the embryos develop *ex utero*, zebrafish are also an excellent model for studying pharmacology and toxicology in early developmental stages. Finally, the zebrafish is a vertebrate species, and many of its genes and metabolic systems are highly conserved with humans; indeed, 80% of genes and expressed sequence tags (ESTs) are present in conserved synteny groups between fish and humans (Barbazuk et al., 2000).

Here, we systematically review the major findings obtained from zebrafish studies of trace element homeostasis, with a focus on iron, zinc, copper, selenium, manganese, and iodine. We also performed a homology analysis of trace mineral transporters in fish, mice and humans, and we summarize the available zebrafish mutant models in the field. This review demonstrates that zebrafish are an ideal experimental tool for investigating novel mechanisms of trace mineral metabolism and for improving therapeutic approaches for treating mineral imbalance-related diseases.

## Zebrafish and iron metabolism

### Overview of iron metabolism

**Figure 1:**
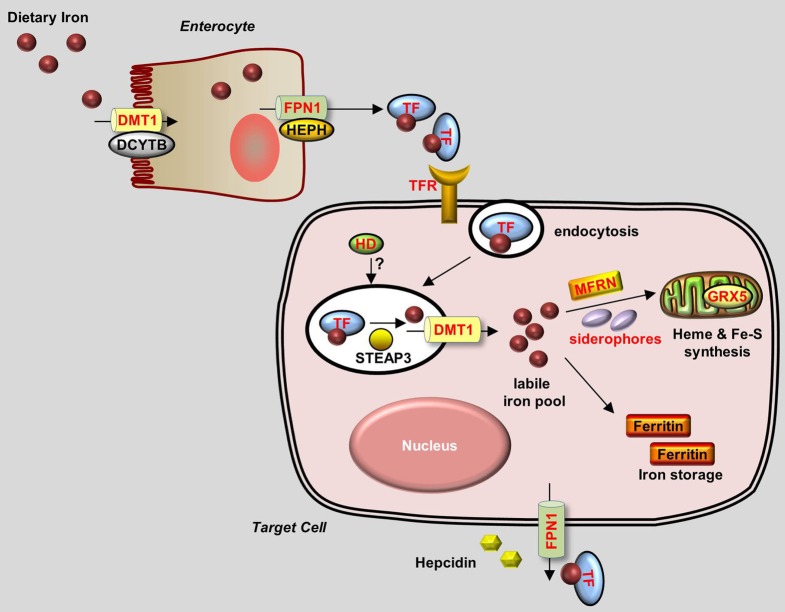
**Generalized overview of iron metabolism in vertebrate cells**. Dietary iron is absorbed by enterocytes through the concerted activity of the reductase DCYTB and the transporter DMT1. Iron is then oxidized by HEPH and exits the enterocytes through the iron exporter FPN1. Iron is transferred as a complex with Transferrin (TF) in the bloodstream and is delivered to target cells that express Transferrin receptors (TFRs) on their plasma membrane. TF-Iron-TFR complexes are then endocytosed. In the endosome, iron is released from TF by STEAP3 and then transported out of the endosome through DMT1. The cytoplasmic iron then enters the labile iron pool and is delivered by MFRN and siderophores to the mitochondria to be used for the synthesis of heme and Fe-S clusters. Excess iron is stored in Ferritin. Iron leaves the cell through FPN1, the plasma expression of which is negatively regulated by Hepcidin. Proteins for which zebrafish knockout and/or knockdown models are available are written in red.

Iron is present in nearly all living organisms. As an essential component of heme and iron-sulfur cluster–containing proteins, iron plays a central role in many biological activities, including oxygen transport, cellular respiration, and DNA synthesis (Muckenthaler and Lill, 2012). Of all the trace elements, the iron homeostasis system is one of the best characterized, primarily because of iron's role in erythropoiesis and its causative relationships with iron-deficiency anemia and hematochromatosis. To date, many major proteins involved in the uptake, transport, storage and release of iron have been identified (Figure 1).

Under normal conditions, dietary iron is absorbed by enterocytes through Divalent Metal Transporter 1 (DMT1); from there, it is exported to the circulation through Ferroportin 1 (Fpn1). In the blood, iron is transported in the form of Transferrin (Tf)-Fe^3+^, which is taken up by endocytosis into cells with surface Transferrin receptors (TfRs). Iron in the endosomes is then released to the cytoplasm and delivered to the mitochondria, where it is used to make iron-sulfur (Fe-S) clusters, to synthesize heme, or to be stored as Ferritin. Most of the iron used for producing hemoglobin in erythrocytes is obtained from the recycling iron pool released from senescent red blood cells that are phagocytized by macrophages. Aside from transport and storage proteins, Hepcidin—a peptide hormone released by the liver—plays an important role in regulating iron levels by binding to Fpn1 and promoting its internalization. Other factors such as oxidoreductases [e.g., Duodenal Cytochrome b (Dytb), Ceruloplasmin (Cp), Hephaestin (Heph), and STEAP3) and modulatory proteins (e.g., Hemochromatosis (HFE), Hemojuvelin (HJV), Iron Regulatory Protein (IRP) 1/2, and Transmembrane Serine Protease 6 (TMPRSS6)] also play an active role in iron metabolism (Muckenthaler and Lill, 2012; Srai and Sharp, 2012). An overview of the protein homology and expression patterns among fish and mammalian iron-regulating proteins is provided in Table 1.

**Table 1:** **Iron metabolism–related proteins in zebrafish, mice, and humans**.

**Gene/Protein**	**Function/Related diseases**	**Tissue distribution**		**Protein identity (vs. H)**	
				***M%***	***Z%***
*SLC11A2*/DMT1	Intestinal iron absorption, intracellular iron release/Hypochromic microcytic anemia	H	Ubiquitous	89	71
		M	Yolk sac, intestine		
		Z	Blood, gill, gut, lens, liver, YSL		
*SLC40A1*/FPN 1	Cellular iron efflux/Hemochromatosis type 4	H	Duodenum, macrophages, KCs, placenta, kidney	91	68
		M	Placenta, intestine, bone marrow, erythrocytes, liver, spleen		
		Z	CNS, yolk syncytial layer, gill, gut, liver		
*HAMP*/Hepcidin	Cellular iron homeostasis/Hemochromatosis type 2B	H	Liver, heart	59	31^a^/32^b^
		M	Liver, lung, heart		
		Z	*Hamp1*: gut, liver; *hamp2*: N.D.		
*HFE*/HFE	Regulator of Tf-TfR interaction/Hemochromatosis type 1	H	Ubiquitous	68	N.D.
		M	Ubiquitous		
		Z	N.D.		
*HFE2*/HJV	Modulator of hepcidin expression/Hemochromatosis type 2A	H	Ubiquitous	87	44
		M	Skeletal muscle, liver, heart, prostate		
		Z	Skeletal muscle, liver, notochord		
*TF*/Transferrin	Transport iron/atransferrinemia	H	Liver, spinal cord, lung, hypothalamus	72	41
		M	Liver, spinal cord, cerebellum, lung, placenta, ovary, bladder		
		Z	Liver, trunk musculature		
*TFRC*/TFR1	Cellular iron uptake / N.D.	H	Fetal liver, pancreas, muscle, placenta, early erythroid cells	77	44^c^/39^d^
		M	Placenta, intestine, muscle, osteoclasts, microglia, bone marrow, liver, kidney		
		Z	*Tfr1a*: blood, blood island; *tfr1b*: ubiquitous		
*TFR2*/TFR2	Mediates cellular uptake of Tf-bound iron/Hemochromatosis, type 3	H	Liver, early erythroid cells	85	53
		M	Liver, bone, bone marrow		
		Z	Liver		
*TMPRSS6*/TMPRSS6	Iron homeostasis/Iron-refractory iron deficiency anemia	H	Ubiquitous	83	N.D.
		M	Liver		
		Z	N.D.		
*SLC25A37*/Mitoferrin	Mitochondrial iron transport/N.D.	H	Bone marrow, fetal liver, fetal lung, blood, prostate, early erythroid cells	91	69
		M	Umbilical cord, spleen, bone, bone marrow		
		Z	Blood island, lateral plate mesoderm		
*CYBRD1*/DCYTB	Dietary iron absorption/N.D.	H	Ubiquitous	75	58
		M	Ubiquitous		
		Z	N.D.		
*STEAP3*/ STEAP3	Ferric-chelate reductase activity/Anemia, hypochromic microcytic, with iron overload 2	H	Ubiquitous	87	53
		M	Ubiquitous		
		Z	N.D.		
*CP*/Ceruloplasmin	Oxidizes Fe(II) to Fe(III)/aceruloplasminemia	H	Ubiquitous	83	55
		M	Mammary gland, lung, liver, lens		
		Z	Liver, gut, pancreas		
*HEPH*/Hephaestin	Transport of dietary iron from epithelial cells of the intestinal lumen into the circulatory system/N.D.	H	Ubiquitous	86	N.D.
		M	Intestine, stomach, ovary, brown adipose		
		Z	N.D.		
*FTH1*/Ferritin	Store of iron in a soluble and nontoxic state/hemochromatosis, type 5	H	Ubiquitous	92	77^e^/76^f^
		M	Ubiquitous		
		Z	*fth1a*: blood, eye; *fth1b*: N.D.		
*ACO1*/IRP1	Interacts with mRNA to control the levels of iron inside cells/N.D.	H	Ubiquitous	93	82
		M	Ubiquitous		
		Z	Blood		
*IREB2*/IRP2	Iron-responsive element binding/N.D.	H	Ubiquitous	94	65
		M	Ubiquitous		
		Z	Blood		
*FXN*/Frataxin	Regulates mitochondrial iron transport and respiration/Friedreich's ataxia	H	Ubiquitous	73	43
		M	Ubiquitous		
		Z	N.D.		
*GRX5*/Glutaredoxin 5	Involved in the biogenesis of iron-sulfur clusters/pyridoxine-refractory sideroblastic anemia	H	Ubiquitous	94	59
		M	Ubiquitous		
		Z	Blood island, dorsal aorta, heart, liver		

a *hamp1*.

b *hamp2*.

c *tfr1a*.

d *tfr1b*.

e *fth1a*.

f *fth1b*.

*H, human; M, mouse; Z, zebrafish; N.D., Not determined; YSL, yolk syncytial layer; KC, Kupffer cells; CNS, central nervous system*.

Zebrafish absorb waterborne iron via the gastrointestinal tracts and the gills. The fish branchial iron uptake has high- and low-affinity components, with Km of 5.9 nmol/l Fe and Vmax of 2.1 pmol/g·h at low Fe concentration (<40 nmol/1), and a linear manner increase of the uptake rate at higher Fe concentration (40–200 nmol/1) (Bury and Grosell, 2003). Zebrafish branchial iron transport can be inhibited by high level of Cd, but not by other divalent metals such as Zn, Cu, and Mn (Bury and Grosell, 2003). Moreover, low iron diet fed zebrafish exhibited a significant increase in tissue Cd accumulation, suggesting an interaction between Fe and Cd assimilation in fish (Cooper et al., 2006).

### Zebrafish models of iron metabolism identified using forward genetic screens

The Nüsslein-Volhard lab performed two large-scale *N*-ethyl-*N*-nitrosourea (ENU) mutagenesis screens in zebrafish and identified several thousand mutations that affect various aspects of early development (Haffter et al., 1996). Using this strategy with an anemic phenotype as the screening output, several mutant zebrafish lines with defects in iron metabolism have been established; these lines are summarized in Table 2.

**Table 2:** **Iron metabolism–related mouse and zebrafish knockout/knockdown models and their phenotypes**.

**Gene/Protein**	**KO mouse models**	**KO/KD zebrafish models**	**References**
*SLC11A2*/DMT1	Hypochromic microcytic anemia^a,b^	Hypochromic microcytic anemia^c^	^a^Russell et al., 1970; ^b^Gunshin et al., 2005a; ^c^Donovan et al., 2002
*SLC40A1*/FPN1	Embryonic lethality; abnormal iron homeostasis^d,e^	Hypochromic microcytic anemia^f^	^d^Donovan et al., 2005; ^e^Zohn et al., 2007; ^f^Donovan et al., 2000
*HAMP*/Hepcidin	Massive iron accumulation in the liver, pancreas, and heart^g^	N.D.	^g^Lesbordes-Brion et al., 2006
*HFE*/HFE	Increased intestinal iron absorption, elevated hepatic iron load, reduced duodenal iron stores^h,i^	N.D.	^h^Vujic Spasic et al., 2007; ^i^Vujic Spasic et al., 2008
*HFE2*/HJV	Lack of hepcidin expression, severe iron overload^j,k^	N.D.	^j^Huang et al., 2005a; ^k^Niederkofler et al., 2005
*TF*/TF	Hypochromic microcytic anemia, iron-loading in the liver, pancreas, heart, and brain^l^	Hypochromic anemia^m^	^l^Bartnikas et al., 2011; ^m^Fraenkel et al., 2009
*TFRC*/TFR1	Anemia, hydrops fetalis, neurological defects^n^	Hypochromic anemia^o^	^n^Levy et al., 1999; ^o^Wingert et al., 2004
*TFR2*/TFR2	Periportal hepatic iron loading, splenic iron sparing, and elevated serum transferrin saturations ^p^	N.D.	^p^Roetto et al., 2010
*TMPRSS6*/TMPRSS6	Microcytic anemia, female infertility^q,r^	N.D.	^q^Nai et al., 2010; ^r^Nai et al., 2012
*SLC25A37*/MFRN	No hemoglobinization in the yolk sac and heart; die during organogenesis^s^	Hypochromic anemia, erythroid maturation arrest^t^	^s^Troadec et al., 2011; ^t^Shaw et al., 2006
*CYBRD1*/DCYTB	Alterations in liver weight and liver iron content^u^	N.D.	^u^Gunshin et al., 2005b
*STEAP3*/STEAP3	Anemia^v^	N.D.	^v^Ohgami et al., 2005
*CP*/Ceruloplasmin	Iron accumulation in the liver, spleen, brain; iron deficiency anemia, impaired motor coordination^w,x^	N.D.	^w^Harris et al., 1999; ^x^Cherukuri et al., 2005
*HEPH*/Hephaestin	Hypochromic anemia^y^	N.D.	^y^Vulpe et al., 1999
*FTH1*/Ferritin	Embryonic lethality ^z^	N.D.	^z^Darshan et al., 2009
*GRX5*/GRX5	N.D.	Hypochromic anemia^A^	^A^Wingert et al., 2005
*HD*/Huntingtin	N.D.	^*^Hypochromic anemia^B^	^B^Lumsden et al., 2007
*BDH2*/2,5-DHBA	N.D.	^*^Hypochromic anemia^C^	^C^Devireddy et al., 2010
*ARHGEF3*/ARHGEF3	N.D.	^*^Hypochromic anemia^D^	^D^Serbanovic-Canic et al., 2011

* *Knockdown (KD) model*.

#### Ferroportin 1

Although researches had long suspected that an iron exporter is present in enterocytes, this was only confirmed in 2000 by studies performed in the zebrafish mutant *weissherbst* (*weh*) (Donovan et al., 2000). The *weh* mutant line was originally isolated from the 1996 Tübingen screen (Haffter et al., 1996) and was subsequently found to have severe hypochromic anemia phenotypes, including decreased hemoglobin levels, blocked erythroid maturation, and reduced numbers of erythrocytes (Ransom et al., 1996; Donovan et al., 2000). Interestingly, mutant embryos have significantly lower iron levels in their erythroid cells compared with wild-type fish, suggesting a circulatory iron deficiency. Indeed, the reduced hemoglobin level in *weh* mutants can be rescued by intravenous iron-dextran injections, demonstrating that their hypochromia is caused by inadequate iron in the blood (Donovan et al., 2000). To identify the precise location of the gene mutation, chromosomal walking was performed and revealed a premature stop codon in a novel gene named *ferroportin1* (*fpn1*), consistent with a loss-of-function mutation. Importantly, overexpressing *fpn1* in mutant embryos rescued the hypochromia phenotype, suggesting that the *fpn1* gene is causally linked to the disease. *Fpn1* transcripts are present specifically in the zebrafish yolk syncytial layer (YSL), between the developing hematopoietic cells in the intermediate cell mass and the yolk, which contains iron and other nutrients essential for early embryonic development. This specific expression pattern of *fpn1*, together with the iron-deficiency phenotypes observed in *weh* mutants, suggests that the function of the fpn1 protein is to export iron from the yolk into the embryonic circulation. This hypothesis was confirmed by performing an iron efflux assay in a *Xenopus* oocyte expression system, in which oocytes expressing *fpn1* had increased iron efflux (Donovan et al., 2000). Moreover, both mice and humans have homologs of *FPN1* that are highly conserved with the fish *fpn1*, and mammalian *FPN1* is robustly expressed in the placenta, duodenum, and liver, all of which are major sites of iron transport. At the protein level, human FPN1 is concentrated at the basal surface of the syncytiotrophoblasts in the placenta, an organ that is functionally similar to the zebrafish YSL, indicating that human FPN1 plays a role in maternal-fetal iron export. In mice, *Fpn1* is expressed at the basolateral surface of enterocytes, suggesting a role as an intestinal iron transporter (Donovan et al., 2000). This study serves as a prime example as how genetic screens in zebrafish can lead to the identification of essential novel genes. Nevertheless, Fpn1 remains the only iron exporter that has been identified in all eukaryotic organisms.

Shortly after these findings in the zebrafish *weh* mutants were reported (Donovan et al., 2000), two groups independently cloned *Fpn1* (also called *Ireg1* or *Mtp1*) from mouse duodenal epithelial cells and a mouse mRNA library (Abboud and Haile, 2000; McKie et al., 2000). Both studies confirmed the essential role of Fpn1 in iron export and the regulation of iron homeostasis. Mutations in *SLC11A3*, the human homolog of *FPN1*, were later identified as causing autosomal dominant hemochromatosis, a disorder characterized by iron overload and multiple organ damage (Montosi et al., 2001; Njajou et al., 2001).

The effect of Fpn1 deficiency on the adult zebrafish system was examined in further detail. Although *weh* homozygotes are embryonic lethal and usually die 7–14 days post-fertilization (dpf), repeated intravenous injections of iron-dextran enables the mutants to reach adulthood (Donovan et al., 2000; Fraenkel et al., 2005). These rescued fish are normal until 6 months of age, but develop hypochromic blood by 12 months. Compared with iron-injected wild-type fish, the rescued mutants had increased iron staining in the kidney macrophages at 12 months of age, as well as increased staining in the intestinal villi at 6 and 12 months, suggesting that the *fpn1* mutation impairs iron export in these tissues. The iron-rescued *weh* mutants also has hepatic iron overload, with particularly high iron levels in the liver Kupffer cells (Fraenkel et al., 2005). The role of Fpn1 in iron mobilization from enterocytes, hepatocytes, and macrophages was confirmed by studies using adult tissue-specific *Fpn1* knockout mice (Zhang et al., 2011, 2012). Hepcidin, a peptide hormone secreted by the liver, was found to regulate iron by triggering the internalization of Fpn1 and inhibiting iron efflux (Nemeth et al., 2004). Hepcidin is also conserved in fish, and injecting zebrafish embryos with iron causes a significant increase in endogenous *hepcidin* expression. Moreover, a similar iron-stimulated increase in *hepcidin* expression occurs in *weh* mutant embryos, suggesting that *hepcidin* expression is independent of Fpn1's normal function as an iron exporter (Fraenkel et al., 2005).

#### Dmt1

The protein DMT1 (also called DCT1 and Nramp2), which contains 12 transmembrane domains, was originally isolated in the rat duodenum as a divalent ion transporter that is upregulated by dietary iron deficiency (Gunshin et al., 1997). Shortly after its discovery, two mammalian hypochromic anemia models—the *mk*/*mk* mouse and the Belgrade rat—were found to carry DMT1 mutations (Fleming et al., 1997, 1998). The *chardonnay* (*cdy*) zebrafish mutant revealed a conserved role of DMT1 in zebrafish iron metabolism (Donovan et al., 2002). The *cdy* mutant is a hypochromic, microcytic anemia model with reduced hemoglobin expression and delayed erythrocyte maturation. The *cdy* mutant has a premature stop codon in the zebrafish homolog of *dmt1*, resulting in a severely truncated protein. The zebrafish homolog of DMT1 is 73% identical to human and mouse DMT1 homologs, and its transcripts are concentrated both in erythroid cells and in the intestine. Direct evidence of the role of zebrafish DMT1 in iron transport came from experiments using a mammalian cell line; cells that overexpressed wild-type zebrafish *dmt1* took up nearly 10 times the amount of iron as non-transfected control cells, whereas the truncated protein produced by the *cdy* mutation was non-functional. Interestingly, unlike the *weh* mutants, which die during early development (Donovan et al., 2000), the anemic *cdy* homozygotes survive and reach adulthood. The viability of *cdy* fish may be attributed to additional pathways for iron absorption (Donovan et al., 2002). Of clinical relevance, the first identified human mutation in *DMT1* was reported to cause symptoms that include severe hypochromic microcytic anemia and iron overload (Mims et al., 2005). One possible explanation for the excess iron in these patients is the presence of an alternate iron absorption route in the duodenum, thereby bypassing DMT1 (Mims et al., 2005).

#### Tfr1

Transferrin receptor 1 (Tfr1) is a membrane-bound protein that facilitates iron uptake by binding to the iron carrier Transferrin. Tfr1 was identified as being essential for erythropoiesis and embryonic development in a *Tfr1*-knockout mouse model (Levy et al., 1999). *Tfr1*^−/−^ mice develop anemia, have retarded growth and neurological defects, and die during embryogenesis (Levy et al., 1999). Four different zebrafish *chianti* (*cia*) mutants were identified with various degrees of hypochromic anemia and defective erythroid differentiation (Haffter et al., 1996; Wingert et al., 2004), and positional cloning revealed that *cia* alleles are missense and splicing mutations. During early development, *tfr1a* transcripts are expressed specifically in erythrocytes. Importantly, cytoplasmic delivery of iron by microinjection at the 1-cell stage—but not intravenous iron injections—can rescue the hypochromia phenotypes of *cia* mutants, indicating that the *tfr1a* mutation prevents erythrocytes from taking up and utilizing circulating iron (Wingert et al., 2004). Interestingly, while cloning *tfr1a*, a second *tfr1*-like gene, *tfr1b*, was also identified (Wingert et al., 2004). This gene duplication phenomenon in zebrafish is believed to have occurred as an evolutionary genetic event in teleosts (Amores et al., 1998; Postlethwait et al., 1998). The *tfr1b* gene is expressed ubiquitously throughout embryogenesis. Notably, although overexpressing *tfr1b* partially rescues the anemic phenotypes of *cia* mutants, *tfr1b* morphants (animals in which the gene has been knocked down by injecting morpholino antisense oligonucleotides) have normal hemoglobinization. Nevertheless, *tfr1b* morphants have retarded growth and develop brain necrosis, a phenotype that is similar to the neurologic defects observed in the mouse model (Levy et al., 1999), indicating that *tfr1b* may be involved in iron uptake through non-erythroid tissues (Wingert et al., 2004). Thus, the combined phenotypes of *tfr1a* and *tfr1b* deficient zebrafish embryos appear to recapitulate the entire phenotypic spectrum of *Tfr1*^−/−^ mice. Therefore, the *cia* mutant zebrafish is an ideal model for studying the function of *tfr1* in erythropoiesis without the complication of other developmental abnormalities.

#### Grx5

The *shiraz* (*sir*) zebrafish mutants were originally isolated from the Tübingen 2000 screen consortium; these mutants were later identified as a typical hypochromic anemia disease model with a deletion in the *glutaredoxin 5* (*grx5*) gene which encodes an antioxidant protein (Wingert et al., 2005). Functional studies of *grx5* in *sir* zebrafish revealed a novel connection between heme biosynthesis and iron-sulfur (Fe-S) cluster formation, two primary functions of iron that were previously believed to be independent processes in vertebrates.

Studies in yeast revealed that *GRX5* is required for the mitochondrial synthesis of Fe-S clusters (Rodriguez-Manzaneque et al., 2002). Similar to the yeast GRX5, zebrafish grx5 is also localized primarily in the mitochondria, and expression of the zebrafish *grx5* gene can rescue a *GRX5*-deficient yeast strain, suggesting that the function of *grx5* is evolutionarily conserved. However, the *sir* zebrafish mutants have a hypochromic anemia phenotype, with no changes in their mitochondrial iron content or oxidative stress level (Wingert et al., 2005). With respect to iron metabolism, one key difference between yeast and higher eukaryotes is that in the latter, iron regulatory proteins 1 and 2 (IRP1/2) control intracellular iron levels by binding to Iron Response Elements (IREs) in the 5′-UTR of target gene transcripts, thereby blocking their translation. Importantly, the IRE-binding capacity of IRP1 is negatively regulated by Fe-S clusters. Thus, a possible explanation for the anemic phenotype in *sir* zebrafish mutants is that the *grx5* mutation reduces Fe-S assembly, inappropriately triggering IRP1 activity, which then inhibits the expression of select target genes that are critical for heme biosynthesis. In support of this hypothesis, red blood cells in *sir* zebrafish mutants lack aminolevulinate synthase 2 (ALAS2), the first enzyme in the heme biosynthesis pathway. Moreover, overexpressing an *ALAS2* gene in which the IRE is deleted rescues hemoglobin production in *sir* mutants; in contrast, overexpressing the wild-type *ALAS2* gene does not rescue hemoglobin production. Interestingly, knocking down the expression of IRP1 also rescues the *sir* embryonic phenotype. These compelling results strongly suggest that heme synthesis in vertebrates is regulated via Fe-S cluster levels (Wingert et al., 2005). A conserved role for GRX5 in regulating heme synthesis was additionally confirmed in human patients (Camaschella et al., 2007). The findings obtained from *sir* mutants serve to highlight the advantages of using zebrafish as a vertebrate model system for discovering mechanisms that are not necessarily conserved in lower organisms.

#### Mitoferrin

The role of Mitoferrin (Mfrn) in mitochondrial iron uptake was originally discovered in yeast studies (Foury and Roganti, 2002). MRS3 and MRS4, the yeast homologs of Mfrn, increase the efficiencies of both heme formation and Fe-S biosynthesis, the two key mitochondrial processes that utilize iron (Muhlenhoff et al., 2003). Studies of *frascati* (*frs*) zebrafish mutants further support Mfrn's role as a principal mitochondrial iron importer in vertebrate erythroblasts. *Frs* mutants develop defects such as hypochromic anemia and erythroid maturation arrest (Shaw et al., 2006), and positional cloning identified missense mutations in the *mfrn* gene in all mutant lines. Importantly, overexpressing *mfrn* in *frs* mutants rescued the erythropoiesis deficiency in half of the injected animals, and *mfrn* knockdown morphants mimicked the mutant phenotype, suggesting that *mfrn* is the disease-causing gene in *frs* mutants. Expression array analysis revealed that *mfrn* is highly expressed in the intermediate cell mass (the tissue in which erythropoiesis occurs for early embryos), further supporting *mfrn*'s role in erythroid heme synthesis. Similar to yeast *MRS3/4*, when overexpressed in transfected mammalian cell lines, zebrafish *mfrn* localizes to the mitochondria (Shaw et al., 2006). To investigate the function of mammalian *Mfrn* further, an *Mfrn^−/−^* mouse hematopoietic cell line was established. These cells have impaired terminal erythroid maturation and an inability to incorporate iron into heme proteins. Furthermore, mouse *Mfrn* rescues the phenotype in zebrafish *frs* mutants, and fish *mfrn* restores the activity of an *MRS3/4*-deficient yeast strain (Shaw et al., 2006). Taken together, these results suggest that Mfrn's role in mitochondrial iron uptake is evolutionarily conserved among eukaryotes.

#### Transferrin-a

Zebrafish *gav* mutant strains carry mutations in their *transferrin-a* (*tf-a*) gene, which encodes the principal iron carrier in all vertebrate organisms (Fraenkel et al., 2009). *Gav* mutants develop severe hypochromic anemia. Importantly, this phenotype can be phenocopied by injecting *tf-a* morpholinos into embryos, and it can be rescued by overexpressing *tf-a*. Together, these findings confirm that *tf-a* is the disease-causing gene. Homozygous *gav* mutants are generally embryonic lethal and die at approximately 14 dpf (Fraenkel et al., 2009). In humans, genetic mutations in transferrin cause congenital hypotransferrinemia, a rare disease with features that are strikingly similar to the phenotype in *gav* fish, including hypochromic anemia and premature death (Hayashi et al., 1993; Goldwurm et al., 2000). Thus, the zebrafish *gav* mutant serves as an ideal model for studying human hypotransferrinemia. In addition, Fraenkel and colleagues examined *hepcidin* expression in the *gav* mutant, as well as several previously established zebrafish mutants with iron metabolism defects. In 2-dpf zebrafish embryos (the stage in which endogenous *hepcidin* expression is least affected by environmental stimuli), the number of *hepcidin* transcripts was measured in various mutants and morphants either with or without iron injection. The results suggest that both *tf-a* and *tfR2* are required for hepcidin expression, whereas *tfR1a* and *dmt1* are required for increasing *hepcidin* expression in response to iron loading (Fraenkel et al., 2009). The primary role of Tf in driving *hepcidin* expression is further supported by studies performed using a mouse model of hypotransferrinemia (Bartnikas et al., 2011).

### Zebrafish as a reverse genetics tool in iron metabolism studies

In addition to helping identify novel iron transporters and metabolic mechanisms via forward genetic screens, zebrafish have also been used as a reverse genetics tool for increasing our understanding of the iron homeostasis system.

#### Gene knockdown/knockout

Gene knockdown/knockout techniques are used as a primary step in investigating the unknown biological functions of a given gene. In this approach, antisense morpholino oligonucleotides bind to the targeted gene transcript, thereby inhibiting the gene's expression by blocking the initiation of translation or by modifying pre-mRNA splicing. Because zebrafish develop *ex utero*, microinjection-mediated gene suppression is used widely among zebrafish researchers.

***Hd***. Studies performed using hd zebrafish morphants revealed a novel role for Huntingtin (Htt, a protein linked to Huntington's disease) in the utilization of iron by erythrocytes (Lumsden et al., 2007). Although it has been known for more than two decades that Huntington's patients carry an expanded CAG repeat in the coding region of the HD gene (Huntington's Disease Collaborative Research Group, 1993), the normal function of Htt remains unclear. In order to explore the biological roles of HD, hd knockdown morphant zebrafish were created (Lumsden et al., 2007). Interestingly, in addition to neurological defects such as brain necrosis, the hd knockdown fish also develop blood hypochromia (characterized by reduced red pigments in the erythrocytes) and decreased hemoglobin staining. Nevertheless, Prussian blue staining revealed that hd morphants have normal iron levels in their red blood cells, suggesting that Tf-Tfr–mediated iron transport and endocytosis are intact in the erythrocytes. Intriguingly, Prussian blue only stains ferric iron that is bound to Tf, but does not stain ferrous iron in hemoglobin. Thus, the hypochromia observed in the hd morphants is likely due to defects that are downstream of Tf-Tfr endocytosis. This hypothesis was tested by injecting iron-dextran directly into the cytoplasm at the 1-cell stage, which circumvents the Tf-Tfr–mediated iron cycle. Using this approach, the hypochromia defects in hd morphants were largely rescued (Lumsden et al., 2007). This important study revealed an interesting role for Htt in making endocytosed iron available for use by the cell; however, the detailed mechanism by which Htt regulates iron release and/or downstream utilization must be explored further.

***Bdh2***. Although the vast majority of intracellular iron is bound by proteins, a small amount of cytoplasmic iron is bound to low-molecular-weight carriers called siderophores, forming a labile iron pool (Breuer et al., 2008). Enterobactin is a classic bacterial siderophore that binds to Lipocalin 24p3, an iron-trafficking protein that functions as the iron-chelating moiety (Yang et al., 2002). In mouse cell cultures, 2,5-dihydroxybenzoic acid (2,5-DHBA) is the iron-binding moiety of the 24p-associated mammalian siderophore, the synthesis of which is catalyzed by 3-hydroxybutyrate dehydrogenase type 2 (Bdh2), a dehydrogenase/reductase family member (Devireddy et al., 2010). In Bdh2-knockdown mouse cells, the intracellular siderophore was depleted, and the cells accumulated abnormally high amounts of free cytoplasmic iron, resulting in elevated levels of ROS. Notably, these cells were also deficient in mitochondrial iron, suggesting that siderophores also participate in the transport of iron from the cytoplasm to the mitochondria. Importantly, bdh2 zebrafish morphants develop hypochromic blood and have reduced hemoglobin levels—but normal globin expression—confirming a defect in mitochondrial heme synthesis (Devireddy et al., 2010). These findings demonstrate that the function of siderophores in regulating intracellular iron homeostasis is conserved from bacteria to vertebrates. In this respect, zebrafish are a convenient model for confirming and extending findings obtained from studying mammalian cell cultures.

***Arhgef3***. Genome-wide association and meta-analysis studies have identified more than 100 independent genetic loci associated with erythrocytes and platelets (Ganesh et al., 2009; Soranzo et al., 2009). Because of its advantages with respect to reverse genetics, the zebrafish model was used to investigate the biological functions of several candidates identified from the meta-analysis. This screen revealed that Rho guanine nucleotide exchange factor 3 (Arhgef3) plays an unexpected role in regulating iron uptake and driving erythroid cell maturation (Serbanovic-Canic et al., 2011). Silencing arhgef3 expression in zebrafish disrupts erythroid differentiation and causes hypochromic erythrocytes, which are indicative of iron-deficiency anemia. Indeed, cytoplasmic iron supplementation significantly rescues the hemoglobinization phenotype in arhgef3 morphants. Moreover, disrupting the arhgef3 target RhoA produces a phenotype that is similar to arhgef3 morphants, and this can also be rescued by cytoplasmic iron injection. The concerted roles of Arhgef3 and RhoA in regulating the internalization of membrane-bound Tf was supported by studies in a human cell line (Serbanovic-Canic et al., 2011).

***In vivo validation of cis-regulatory elements in mfrn1 using transgenic fish***. In erythroblasts, Mfrn1, and Mfrn2 are solute carriers that import cytoplasmic iron into the mitochondria for heme and Fe-S cluster biogenesis (Shaw et al., 2006; Paradkar et al., 2009). In mice, both the Mfrn1 and Mfrn2 genes contain CpG-rich promoter regions. The cis-regulatory modules (CRMs) in Mfrn1 form a chromatin immunoprecipitation dataset for GATA-1, the primary erythroid transcription factor (Cantor and Orkin, 2002), suggesting that Mfrn1 is transcriptionally regulated by GATA-1 via binding at CRM regions. Though quite compelling, these bioinformatics results still needed to be validated functionally at the systemic level, and transgenic fish were a convenient tool for this purpose. Mfrn1 transcripts are concentrated primarily in hematopoietic tissues, whereas mfrn2 transcripts are expressed throughout the central nervous system and in somites (Shaw et al., 2006). Transgenic zebrafish expressing GFP-tagged mouse Mfrn1 or Mfrn2 promoter sequences were generated to study the expression of these genes. When expressed in fish, the Mfrn2 promoter is expressed in a pattern similar to the endogenous mfrn2 expression pattern, suggesting that the promoter functions appropriately. However, the tagged Mfrn1 promoter failed to drive detectable GFP expression in fish, suggesting the need for other transcriptional regulatory elements (Amigo et al., 2011). Interestingly, injecting two of the three predicted mouse Mfrn1 CRMs yielded transient transgenic fish that expressed GFP in the same tissues as endogenous mfrn1. The specific expression pattern was refined further in transgenic fish carrying a construct that contains a CRM linked with the CpG-rich promoter. Thus, the critical role of CRMs in regulating Mfrn1 expression has been demonstrated in vivo (Amigo et al., 2011). Moreover, the critical role for GATA-1 in regulating Mfrn1 transcription was confirmed by the finding that the Mfrn1-specific expression pattern was abolished after the GATA-1 core binding sites were mutated in the CRMs of the transgenic fish. Finally, the expression of endogenous mfrn1 was also markedly reduced in GATA-1 zebrafish morphants (Amigo et al., 2011). This seminal study supports the high value of using zebrafish transgenics to complement and validate findings obtained from in silico analyses.

#### Gene overexpression

The *ex utero* development of zebrafish also enables researchers to drive gene overexpression through microinjection. Importantly, the biological mechanisms that underlie dominant-negative gene mutations in mammals can be examined readily in zebrafish using gene overexpression techniques. In humans, *FPN1* mutations have been linked to a form of autosomal dominant hemochromatosis (Pietrangelo, 2004). Interestingly, two distinct clinical phenotypes have been characterized: one phenotype includes iron accumulation in macrophages, low transferrin saturation, and iron-limited erythropoiesis, whereas the other phenotype includes iron accumulation in hepatocytes and high transferrin saturation. The diversity of these clinical traits can be explained by the different natures of the underlying *FPN1* mutations (De Domenico et al., 2006). Mutations that cause a defect in FPN1's cell-surface localization or iron export capacity cause iron loading in macrophages, whereas mutations that impair FPN1's sensitivity to Hepcidin (thus impeding FPN1 incorporation) cause iron accumulation in hepatocytes (De Domenico et al., 2005, 2006; Schimanski et al., 2005). Nevertheless, the clinical complexity of the long-term disease process makes it difficult to determine the precise nature of a given *FPN1* mutation. Overexpressing mutant *FPN1* alleles in zebrafish is a high-throughput approach for identifying the functional effects of many mutations (De Domenico et al., 2007). The cDNA of wild-type mouse *Fpn1* or previously identified human and mice *Fpn1* mutants was injected into 1-cell stage zebrafish embryos. Expressing either wild-type *Fpn1* or the N144H *Fpn1* mutant (a Hepcidin-irresponsive protein) had no effect on endogenous hemoglobinization. However, overexpressing the H32R *Fpn1* mutant (which has defective Fpn1 membrane localization) or the N174I *Fpn1* mutant (which is transport-deficient) led to severe defects in hemoglobin synthesis, and these hemoglobinization defects were rescued by intravenous iron injections, suggesting the existence of iron-deficiency erythropoiesis in these mutant-expressing embryos (De Domenico et al., 2007). This study demonstrates nicely that the functional consequences of mammalian *Fpn1* mutations can be studied rapidly and effectively, and it supports the important role of zebrafish as a valuable vertebrate model in functional studies of dominant-negative mutations.

## Zebrafish and zinc metabolism

### Overview of zinc metabolism

After iron, zinc is the second-most abundant trace mineral in humans. Zinc is essential for the activity of more than 300 enzymes and for maintaining the structural integrity of nearly 2000 transcription factors. Thus, zinc plays a critical role in cellular homeostasis, the immune response, oxidative stress, apoptosis, and aging (Stefanidou et al., 2006; Prasad, 2012). Zinc deficiency can cause a wide range of clinical defects, including growth retardation, hypogonadism, rough skin, weakened immunity, and neurosensory and cognitive disorders (Prasad, 2012). Although zinc is a redox-inactive metal, it can be toxic (albeit less toxic than iron and copper), and both acute and chronic forms of zinc poisoning have been reported to cause hematopoietic abnormalities, altered lipoprotein metabolism, and impaired immune function (Stefanidou et al., 2006).

**Figure 2:**
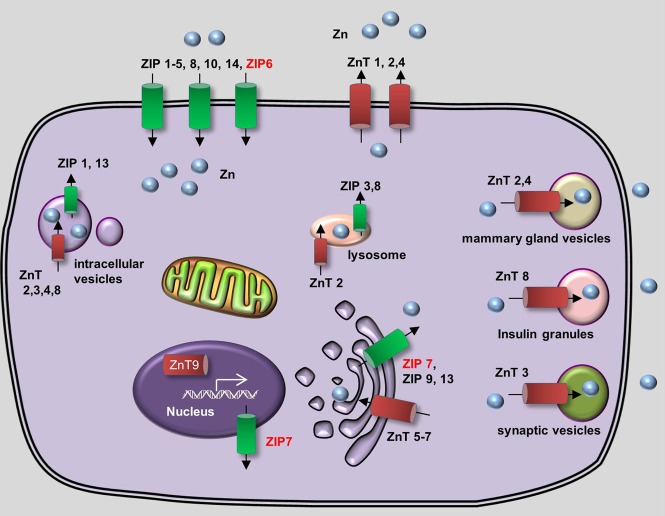
**Generalized overview of zinc metabolism in vertebrate cells**. Zinc Transporters (ZnTs) downregulate intracellular zinc levels by exporting zinc through the plasma membrane (ZnT1, ZnT2, and ZnT4) or by transporting zinc into various intracellular compartments, including lysosomes (ZnT2), the Golgi apparatus (ZnT5-7), mammary gland vesicles (ZnT2 and ZnT4), insulin granules (ZnT8), and synaptic vesicles (ZnT3). In addition, ZnT9 can translocate to the nucleus, where it regulates target gene transcription. Zrt- and Irt-like proteins (ZIPs) upregulate cytoplasmic zinc levels by importing extracellular zinc (ZIP1–6, ZIP8, ZIP10, and ZIP14) and release zinc from intracellular vesicles (ZIP1 and ZIP13), lysosomes (ZIP3 and ZIP8), the Golgi apparatus (ZIP7, ZIP9, and ZIP13) and the nucleus (ZIP7). Proteins for which zebrafish knockout/knockdown models are available are written in red.

*In vivo*, zinc homeostasis relies primarily on the zinc transporter family, which in mammals contains 10 Zinc Transporter proteins (ZnTs, or SLC30) and 14 Zrt- and Irt-like proteins (ZIPs, or SLC39) (Huang and Tepaamorndech, 2013; Jeong and Eide, 2013). ZnTs are zinc exporters that facilitate the efflux of zinc from cells and/or into intracellular vesicles, whereas ZIPs increase intracellular zinc concentration by driving the uptake of extracellular zinc and/or the release of vesicular zinc into the cytoplasm. The concerted actions of ZnTs and ZIPs maintain the balance of intracellular zinc and deliver zinc to its appropriate protein partners (Figure 2). Metallothioneins (MTs) are a group of low-molecular-weight metal-binding proteins that also have a high affinity for binding zinc. MTs play an important regulatory role in zinc metabolism, possibly by competing with—or supplying zinc to—a variety of transporter proteins (Vasak and Hasler, 2000; Chasapis et al., 2012).

### Zinc homeostasis is highly conserved between zebrafish and mammals

The system that regulates zinc metabolism is highly conserved between zebrafish and mammals. The zebrafish *zip1* gene was cloned from the zebrafish gill, an ion-transporting epithelium that absorbs minerals from the surrounding water (Qiu et al., 2005). The Zip1 protein is conserved both structurally and functionally with its mammalian homologs. *Zip1* transcripts are expressed ubiquitously in zebrafish embryos, with the highest expression in the ovaries. As with human *ZIP1* (Gaither and Eide, 2001), overexpressing zebrafish *zip1* significantly increases zinc uptake. Interestingly, because Zip1 does not increase zinc influx at high extracellular zinc concentrations, it has no effect on the maximum endogenous rate of zinc uptake (Qiu et al., 2005). This ceiling effect might be due to the fact that zinc regulates the cellular localization of Zip1. Studies in mice suggest that the extracellular zinc concentration mediates the cellular localization of Zip1 through an endocytosis-mediated pathway (Wang et al., 2004). A second zinc importer in teleosts, *zip2*, was cloned from the gill of a pufferfish species (*Takifugu rubripes*) and plays a role in mediating zinc uptake (Qiu and Hogstrand, 2005).

A systematic bioinformatics data-mining approach identified the zebrafish zinc transporter genes from two previously released zebrafish databases (Zv4 Ensembl 31 and Zv5 Ensembl 34), and these genes were phylogenetically assigned to mammalian orthologs (Feeney et al., 2005). To date, eight ZnT members (ZnT1, ZnT2, and ZnT4–9) and 11 ZIP members (ZIP1, ZIP3, ZIP4, ZIP6–11, ZIP13, and ZIP14) have been identified in zebrafish. Interestingly, the teleost ZIP8 sequence differs from mammalian ZIP8 orthologs to a larger degree than other ZIP genes. Studies of gene expression patterns revealed that the ovaries and intestine—the two organs that have the most dynamic nutrient metabolism—have the highest expression of zinc transporters (Feeney et al., 2005). The expression level of each zinc transporter has been examined during zebrafish embryogenesis under normal maternal zinc conditions (Ho et al., 2012). The results showed that despite a relatively constant level of endogenous zinc during embryonic development from fertilization through 120 h post-fertilization (hpf), zinc transporters are differentially expressed throughout this period. Nearly all zinc transporters have their highest expression at 120 hpf, with the exception of ZNT8, which peaks at 48 hpf (Ho et al., 2012). The release of the latest zebrafish database (Zv9 Ensembl 73) has further increased our knowledge of zebrafish genome, confirming the existence of zebrafish ZNT10 and ZIP5 in zebrafish. The homology of zinc transporters among zebrafish, mice and humans is summarized in Table 3. An influence of Zn on the uptake and circulatory influx of Cd has been reported in fish, suggesting that the uptake of Zn and Cd occurs through common pathways (Wicklund Glynn, 2001).

**Table 3:** **Zinc metabolism–related proteins in zebrafish, mice, and humans**.

**Gene/Protein**	**Function/Related diseases**	**Tissue distribution**		**Protein identity (vs. H)**	
				***M%***	***Z%***
*SLC30A1*/ZNT1	Plasma Zn exporter/N.D.	H	Ubiquitous	86	61^a^/54^b^
		M	Ubiquitous		
		Z	N.D.		
*SLC30A2*/ZNT2	Plasma Zn exporter, transport Zn into mammary gland vesicles/Transient neonatal Zn deficiency	H	Ubiquitous	79	N.D.
		M	Adipose, placenta, intestine, prostate, pancreas, kidney, testis, mammary gland		
		Z	Hindbrain, neurons, spinal cord		
*SLC30A3*/ZNT3	Transport Zn into synaptic vesicles/N.D.	H	Ubiquitous	89	N.D.
		M	Amygdala, cerebral cortex, hippocampus, spinal cord, testis, pancreas		
		Z	N.D.		
*SLC30A4*/ZNT4	Plasma Zn exporter, transport Zn into mammary gland vesicles/Zn deficiency in milk	H	Ubiquitous	92	53
		M	Lacrimal gland, mammary gland, placenta, intestine		
		Z	N.D.		
*SLC30A5*/ZNT5	Plasma Zn exporter, transport Zn into Golgi/N.D.	H	Ubiquitous	95	78
		M	Ubiquitous		
		Z	Ubiquitous		
*SLC30A6*/ZNT6	Transport Zn into Golgi/N.D.	H	Ubiquitous	91	12
		M	Ubiquitous		
		Z	Ubiquitous		
*SLC30A7*/ZNT7	Transport Zn into Golgi/N.D.	H	Ubiquitous	96	79
		M	Ubiquitous		
		Z	CNS, notochord		
*SLC30A8*/ZNT8	Transport Zn into insulin granules/Diabetes mellitus	H	Pancreatic islet	81	53
		M	Ubiquitous		
		Z	Ubiquitous		
*SLC30A9*/ZNT9	Plasma Zn exporter, transcriptional regulation in nucleus/N.D.	H	Ubiquitous	89	73
		M	Ubiquitous		
		Z	Ubiquitous		
*SLC30A10*/ZNT10	Cation transporter/Hypermanganesemia with dystonia, polycythemia, and cirrhosis	H	Ubiquitous	80	46
		M	Stomach, intestine, prostate, liver, amygdala, cerebral cortex		
		Z	N.D.		
*SLC39A1*/ZIP1	Plasma Zn importer, Zn release from vesicles/N.D.	H	Ubiquitous	94	N.D.
		M	Ubiquitous		
		Z	Brain, eye, gill, heart, integument, kidney, musculature system, neural crest, ovary		
*SLC39A2*/ZIP2	Plasma Zn importer/N.D.	H	Ubiquitous	78	N.D.
		M	Ubiquitous		
		Z	N.D.		
*SLC39A3*/ZIP3	Plasma Zn importer, Zn release from lysosomes/N.D.	H	Ubiquitous	84	N.D.
		M	Ubiquitous		
		Z	Ubiquitous		
*SLC39A4*/ZIP4	Plasma Zn importer/acrodermatitis enteropathica	H	Ubiquitous	72	N.D.
		M	Lung, placenta, uterus, ovary, stomach, intestine, liver		
		Z	Ubiquitous		
*SLC39A5*/ZIP5	Plasma Zn importer/N.D.	H	N.D.	84	37
		M	Pancreas, intestine, stomach, placenta, kidney		
		Z	N.D.		
*SLC39A6*/ZIP6	Plasma Zn importer/N.D.	H	Ubiquitous	88	43
		M	Ubiquitous		
		Z	N.D.		
*SLC39A7*/ZIP7	Zn release from Golgi and the nucleus/N.D.	H	Ubiquitous	87	54
		M	Ubiquitous		
		Z	Brain, eye, forebrain, gill, muscle, optic cup, retina		
*SLC39A8*/ZIP8	Plasma Zn importer at the onset of inflammation/N.D.	H	Ubiquitous	89	54
		M	Ubiquitous		
		Z	Ubiquitous		
*SLC39A9*/ZIP9	Zn release from Golgi/N.D.	H	Ubiquitous	93	82
		M	Ubiquitous		
		Z	Ubiquitous		
*SLC39A10*/ZIP10	Plasma Zn importer / N.D.	H	Ubiquitous	87	47
		M	Ubiquitous		
		Z	Anterior axial hypoblast, gill, hatching gland, kidney, pigment cells, polster		
*SLC39A11*/ZIP11	Cation transport/N.D.	H	Ubiquitous	89	67
		M	Ubiquitous		
		Z	Ubiquitous		
*SLC39A12*/ZIP12	Cation transport / N.D.	H	Ubiquitous	78	N.D.
		M	Spinal cord, hypothalamus, retinal pigment, ciliary bodies		
		Z	N.D.		
*SLC39A13*/ZIP13	Zn release from Golgi and vesicles/Ehlers-Danlos syndrome-like spondylocheirodysplasia	H	Ubiquitous	91	53
		M	Ubiquitous		
		Z	Ubiquitous		
*SLC39A14*/ZIP14	Plasma Zn importer/N.D.	H	Smooth muscle, pancreas islet, liver, lung, intestine	83	67
		M	Ubiquitous		
		Z	Notochord, olfactory placode, otic placode, presumptive telencephalon, somite		

a *slc30a1a*.

b *slc30a1b*.

*H, human; M, mouse; Z, zebrafish; N.D., not determined; CNS, central nervous system*.

### Zebrafish models of zinc metabolism

The current zebrafish and mouse models available for studying zinc metabolism are listed in Table 4.

**Table 4:** **Zinc metabolism–related mouse and zebrafish knockout/knockdown models and their phenotypes**.

**Gene/Protein**	**KO mouse models**	**KO/KD zebrafish models**	**References**
SLC30A1/Znt1	Embryonic lethality^a^	N.D	^a^Andrews et al., 2004
SLC30A3/Znt3	Age-dependent deficits in learning and memory^b,c^	N.D	^b^Cole et al., 1999; ^c^Adlard et al., 2010
SLC30A4/Znt4	Zinc-deficient milk, otolith degeneration, impaired motor coordination, alopecia, dermatitis^d^	N.D	^d^Huang and Gitschier, 1997
SLC30A5/Znt5	Growth retarded, skeletal defects^e^	N.D	^e^Inoue et al., 2002
SLC30A7/Znt7	Reduction in body fat accumulation^f^	N.D	^f^Huang et al., 2007
SLC30A8/Znt8	Reduced islet zinc levels, circulating insulin levels, and glucose stimulated insulin secretion^g,h^	N.D	^g^Nicolson et al., 2009; ^h^Pound et al., 2009
SLC39A1/Zip1	Abnormal development^i^	N.D	^i^Dufner-Beattie et al., 2006
SLC39A2/Zip2	Retarded growth^j^	N.D	^j^Peters et al., 2007
SLC39A3/Zip3	No obvious abnormalities^k^	N.D	^k^Dufner-Beattie et al., 2005
SLC39A4/Zip4	Embryonic lethality^l,m^	N.D	^l^Dufner-Beattie et al., 2007; ^m^Geiser et al., 2012
SLC39A6/Zip6	N.D	^*^Embryonic lethality, shortened anterior-posterior axis^n^	^n^Yamashita et al., 2004
SLC39A7/Zip7	N.D	^*^Embryonic lethality, curved notochord, reduced eye size^o^	^o^Yan et al., 2012
SLC39A13/Zip13	Skeletal abnormalities and dental abnormalities^p^	N.D	^p^Fukada et al., 2008
SLC39A14/Zip14	Decreased body size, torticollis, reduced bone volume, scoliosis, impaired fasting gluconeogenesis, decreased hepatic zinc level^q,r^	N.D	^q^Hojyo et al., 2011; ^r^Beker Aydemir et al., 2012

* *Knockdown (KD) model*.

#### Zip6

Zip6 is a member of the LIV-1 subfamily of ZIP zinc transporters, which in humans consists of nine ZIP members that contain a highly conserved metalloprotease motif. Importantly, LIV-1 is regulated by estrogen and has been implicated in metastatic breast cancer (Taylor, 2000), although how LIV-1 mediates cancer metastasis is unclear. Studies using Zip6 (LIV1) zebrafish morphants have been instrumental in addressing this problem (Yamashita et al., 2004); the zebrafish *zip6* cDNA was cloned by subtraction screening. Interestingly, the expression pattern of endogenous *zip6* mimics the expression of *stat3*, an important player in the epithelial-mesenchymal transition (EMT) during gastrulation, organogenesis, wound-healing, and cancer progression (Sano et al., 1999; Yamashita et al., 2002). Notably, the expression of *zip6* is abolished in *stat3* zebrafish morphants, suggesting that *zip6* is downstream of *stat3*. The ability of *stat3* to transcriptionally regulate *zip6* was confirmed in studies using mouse and human cell lines (Yamashita et al., 2004). The role of *zip6* in early embryonic development was assessed further in *zip6*-depleted zebrafish embryos. By the end of gastrulation, these *zip6* morphants have malpositioned heads and a shortened anterior-posterior axis, although early cell-fate specification was not affected, suggesting that *zip6* plays a critical role in cell migration during gastrulation. Cell tracing and cell transplantation assays further support the cell-autonomous role of Zip6 in the migration of mesendodermal cells (Yamashita et al., 2004). Phenotypic analyses of *zip6* morphants revealed that cell-cell adhesion was not downregulated as occurs normally, thus resulting in severe perturbations in cell migration. These same defects were also observed in zebrafish morphants in which the zinc-finger protein Snail, a master regulator of EMT, is knocked down (Batlle et al., 2000; Cano et al., 2000). Moreover, the *in vivo* activity of Zip6 is dependent on Snail; Zip6 regulates the nuclear translocation of Snail (Yamashita et al., 2004). This was the first study to use multiple zebrafish knockdown models to establish a molecular link between Stat3, Zip6, and Snail during EMT.

#### Zip7

Zip7 is also a member of the LIV-1 subfamily of zinc transporters. Studies using mammalian cell lines suggest that human ZIP7 plays a role in elevating cytoplasmic zinc concentrations by transporting zinc from the Golgi apparatus to the cytoplasm (Huang et al., 2005b). The systemic expression and function of Zip7 was examined in zebrafish (Yan et al., 2012), and endogenous Zip7 was found to be expressed ubiquitously in early stages of somitogenesis, but becomes concentrated around the retina after 24 hpf. In adult fish, *zip7* is also highly expressed in the eyes and the brain. *Zip7* zebrafish morphants have developmental defects that include a curved notochord and small eyes. Moreover, co-injecting *zip7* mRNA or supplementing the surrounding water with zinc significantly rescues the phenotypic defects in *zip7* morphants, suggesting that the developmental defects are caused specifically by *zip7* knockdown and are closely related to zinc deficiency (Yan et al., 2012). The distribution pattern of zinc was also compared between wild-type embryos and *zip7* morphants; the analysis revealed a significant loss of zinc in the eyes of the *zip7* morphants, and this was rescued by the addition of exogenous zinc. These results suggest that Zip7 plays a critical role in maintaining intracellular zinc levels in the eyes and demonstrate that exogenous zinc supplementation can compensate for Zip7 deletion, possibly through the activity of other zinc-importing pathways. Indeed, in the *zip7* morphants, the expression levels of several zinc transporters are altered, including *zip3, zip6, znt2, znt5*, and *znt6* (Yan et al., 2012). This study revealed the tissue-specific function of Zip7 and nicely illustrates the dynamic interaction between environmental nutrient levels and endogenous transcriptional regulation.

### Zinc-regulated gene expression in zebrafish

Zinc is required for the function of thousands of transcription factors. Fluctuations in environmental zinc levels actively influence an organism's various biological activities through transcriptional regulation. Zebrafish is a convenient model for studying the effect of fluctuating exogenous nutrients on endogenous gene expression, as the nutrient concentration in the surrounding water can be easily manipulated. In fish, the gill is a unique structure comprised of polarized epithelial cells; this configuration is essential for the fish's ability to extract zinc and other minerals directly from the water. Importantly, the zinc transport system in gills is highly conserved with the transport system in mammals.

Recently, two related studies examined the dynamic transcriptome profiles of gills in zebrafish that were subjected to either zinc depletion or zinc supplementation (Zheng et al., 2010a,b). Juvenile zebrafish were exposed for 2 weeks to water that was either zinc-enriched (4 μM), zinc-normal (0.25 μM), or zinc-deficient (0.04 μM). From 14 days of treatment, fish gill samples were collected from each group at multiple time points and processed through microarray analysis in order to measure changes in the transcriptome. In the group that received zinc supplementation, most of the changes in the transcriptome were associated with “transcription factors,” “steroid hormone receptors,” and “development.” Additional data mining suggested that these detected changes in the transcriptome were likely to be induced by only a few key transcription factors, including Mtf1 (the principal regulator of zinc-driven metallothionein expression), Jun, Stat1, Ppara and Gata3, reflecting a process similar to hedgehog and bone morphogenic protein signaling. Moreover, the transcriptional changes tended to slow after seven days of treatment, suggesting that the fish gradually became acclimated to the elevated zinc in the water (Zheng et al., 2010b). In the zinc-deficient group, the most significant transcriptional changes were found in genes associated with “developmental processes,” which account for up to 26% of all regulated genes. The expression levels of genes correlated with diabetes and bone/cartilage development were also significant changed, which is consistent with previously reported biological roles of zinc (Huang and Tepaamorndech, 2013). Several transcription factors were identified as key coordinators of the homeostatic response to zinc depletion, including Hnf4a, Foxl1, Wt1, Nr5a1, and Nr6a1 (Zheng et al., 2010a). Taken together, these two complementary studies present a systemic, longitudinal overview of the complicated changes that occur in the transcriptome under abnormal environmental zinc levels.

The effect of changing environmental zinc levels on the regulation of zinc transporter expression was also studied in zebrafish by examining the expression patterns of zinc transporters in various tissues under zinc-enriched and zinc-deficient conditions (Feeney et al., 2005). The fish's gills and intestine—two major sites of zinc exchange in fish—had the largest differences in zinc transporter expression. In contrast, the expression of zinc transporters in the muscle and liver was affected the least (Feeney et al., 2005). Two studies examined the transcriptional changes of zinc transporters in fish gills in various zinc concentrations, and these studies reported different sets of genes with altered expression. However, both studies observed increased expression of *znt5, zip3*, and *zip10* in the gills under zinc-deficient conditions and reduced expression of *zip10* under zinc-enriched conditions (Feeney et al., 2005; Zheng et al., 2008). Moreover, the inverse relationship between zinc concentration and *zip10* expression may be controlled by metal-responsive clusters in two distinct promoters in the *zip10* gene that have opposing regulatory roles in response to zinc availability; this process is potentially mediated by Mtf-1 (Zheng et al., 2008). Interestingly, studies using mice have also suggested an essential role for Mtf-1 in regulating *Zip10* expression (Wimmer et al., 2005; Lichten et al., 2011).

## Zebrafish and copper metabolism

### Overview of copper metabolism

Copper is an essential nutrient that is present in nearly all living organisms. Similar to iron, copper is a redox-active metal. Copper functions as a key catalytic cofactor in a wide range of enzymes and is therefore essential for many fundamental biological processes, including cellular respiration, free radical detoxification, connective tissue formation, and melanin production. Despite its essential role in biology, excess copper is highly toxic due to its high redox potential. Copper overload leads to the production of ROS such as hydroxyl radicals, and the accumulation of these radicals can cause devastating damage to cellular components, ultimately causing cell death (Pena et al., 1999). In humans, Menkes syndrome and Wilson's disease are genetic diseases that are caused by copper deficiency and copper overload, respectively (Ala et al., 2007; Tumer and Moller, 2010).

**Figure 3:**
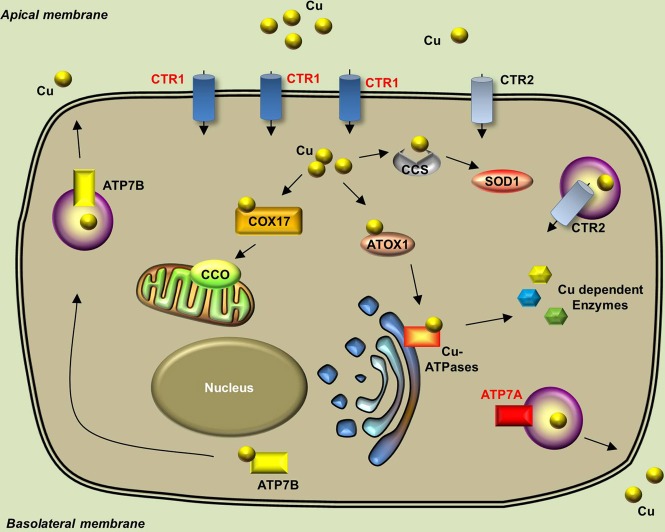
**Generalized overview of copper metabolism in vertebrate cells**. Extracellular copper enters the cell through the high-affinity CTR1 receptor. The CTR2 receptor primarily mediates the release of copper from intracellular vesicles, but is also expressed in low levels in the plasma membrane. Intracellular copper is bound by a variety of copper chaperones and transported to various proteins in the following intracellular sites: COX17 delivers copper to CCO in the mitochondria; CCS delivers copper to cytosolic SOD1; and ATOX1 delivers copper to copper-ATPases in the Golgi apparatus. Copper is secreted from the basolateral and apical sides via ATP7A-mediatedand ATP7B-mediated exocytosis, respectively. Proteins for which zebrafish knockout/knockdown models are available are written in red.

The endogenous copper metabolism system can be divided into three major steps (Figure 3): (i) copper uptake, (ii) intracellular distribution of copper, and (iii) and copper export. The high-affinity copper transporter 1 (CTR1) is the primary player in the uptake of extracellular copper, whereas the low-affinity copper transporter 2 (CTR2) is primarily intracellular and may function to release copper from vesicles. Upon entry into the cell, copper binds to a variety of cytosolic copper chaperones and is then transported to specific subcellular destinations. The three major copper chaperones are Copper chaperone for superoxide dismutase (CCS), Cytochrome c oxidase assembly protein 17 (COX17), and Antioxidant copper chaperone 1 (ATOX1). CCS delivers copper to cytosolic superoxide dismutase 1 (SOD1) to activate its function in mediating superoxide protection. COX17 transports copper to the mitochondria and facilitates its incorporation into Cytochrome c oxidase (CCO), the final enzyme in the respiratory electron transport chain. ATOX1 carries copper to copper-ATPases in the Golgi apparatus, from which copper is then transferred to various cuproenzymes via secretory pathways. The secretion of copper is also dependent on copper-ATPases. ATP7A (the disease gene linked to Menkes syndrome) and ATP7B (the gene that is defective in patients with Wilson's disease) encode two major types of copper exporters. When intracellular copper levels are high, ATP7A and ATP7B are expressed in close proximity to the basolateral and apical membranes, respectively, where they export copper via vesicle-mediated fusion (Lutsenko, 2010). Approximately 95% of copper in the plasma is bound to ceruloplasmin (CP), the principal circulating copper carrier. CP is also a ferroxidase, serving as a molecular link between copper and iron metabolism. The copper metabolism-related proteins in fish and mammals are summarized in Table 5, and the currently available fish and mammalian models are summarized in Table 6.

**Table 5:** **Copper metabolism–related proteins in zebrafish, mice and humans**.

**Gene/Protein**	**Function/Related diseases**	**Tissue distribution**		**Protein identity (vs. H)**	
				***M%***	***Z%***
*SLC31A1*/CTR1	High-affinity Cu transporter/N.D.	H	Ubiquitous	92	72
		M	Ubiquitous		
		Z	Entire embryo; larval brain liver gut; adult gill, liver, gut, ovary		
*SLC31A2*/CTR2	Low-affinity Cu transporter/N.D.	H	Salivary gland, placenta, spinal cord, hypothalamus, blood	77	43
		M	Lacrimal gland, microglia, osteoclasts		
		Z	N.D.		
*ATP6V0D1*/ATP6V0D1	ATP catabolic process; ion transmembrane transport / N.D.	H	Ubiquitous	100	94
		M	Ubiquitous		
		Z	CNS, epiphysis, integument, mucus-secreting cells, neurons, pigment cells, presumptive RPE, kidney, trigeminal placode		
*ATP7A*/ATP7A	Cu-exporting ATPase/Menkes syndrome; occipital horn syndrome; spinal muscular atrophy, distal, X-linked 3	H	Ubiquitous	90	65
		M	Ubiquitous		
		Z	Neural tube, notochord, entire organism		
*ATP7B*/ATP7B	Cu-exporting ATPase/Wilson's disease	H	Ubiquitous	82	62
		M	Ubiquitous		
		Z	Liver		
*COX17*/COX17	Cu chaperone / N.D.	H	Ubiquitous	92	75
		M	Ubiquitous		
		Z	Lens, myotome, pectoral fin musculature, liver, gill		
*CCS*/CCS	Cu chaperone/N.D.	H	Liver, early erythroid cells	87	68
		M	Liver, kidney		
		Z	Ubiquitous		
*ATOX1*/ATOX1	Cu chaperone / N.D.	H	Ubiquitous	85	69
		M	Ubiquitous		
		Z	N.D.		

*H, human; M, mouse; Z, zebrafish; N.D., not determined; CNS, central nervous system; RPE, retinal pigment epithelium*.

**Table 6:** **Copper metabolism–related mouse and zebrafish knockout/knockdown models and their phenotypes**.

**Gene/Protein**	**KO mouse models**	**KO/KD zebrafish models**	**References**
*SLC31A1*/CTR1	Embryonic lethality; decreased copper levels in the blood and several organs^a,b^	^*^Embryonic lethality; cell death in the neural system^c^	^a^Lee et al., 2001; ^b^Nose et al., 2006; ^c^Mackenzie et al., 2004
*ATP6V0D1*/ATP6V0D1	Mortality; premature aging^d^	Embryonic lethality; pigment loss with copper deprivation^e^	^d^Miura et al., 2003; ^e^Madsen and Gitlin, 2008
*ATP7A*/ATP7A	Perturbed copper metabolism^f,g^	Pigment loss; abnormal skeletal and notochord development^h^	^f^Schlief et al., 2006; ^g^Niciu et al., 2007; ^h^Mendelsohn et al., 2006
*ATP7B*/ATP7B	Copper accumulation in various organs, primarily the liver, kidney, and brain; a form of liver cirrhosis^i^	N.D.	^i^Buiakova et al., 1999
*COX17*/COX17	Retarded growth^j^	N.D.	^j^Takahashi et al., 2002
*CCS*/CCS	Increased sensitivity to paraquat and reduced female fertility^k^	N.D.	^k^Wong et al., 2000
*ATOX1*/ATOX1	Impaired intracellular copper trafficking and postnatal mortality, retarded growth, hypoactivity, loose skin, hypopigmentation, seizures^l^	N.D.	^l^Hamza et al., 2001

* *Knockdown model*.

### Zebrafish models of copper metabolism

#### Identifying copper-deficient phenotypes in zebrafish

Unlike iron, which is directly related to anemia and hemochromatosis, many trace minerals do not cause specific phenotypes when they are deficient or in excess. The identification of copper-related phenotypes is essential for identifying animal models to study mineral imbalance. A spectrum of distinct developmental abnormalities was linked to copper deficiency through a chemical genetic screen (Mendelsohn et al., 2006). Copper has been proposed to play a role in melanin formation through the activity of tyrosinase, a copper-containing oxidase (Rawls et al., 2001). Using copper-induced reversible depigmentation as a screening output, a library of small molecules was evaluated for their role in interfering with copper metabolism. Notably, in addition to pigment loss, a specific combination of other abnormalities was observed in all of the molecule-treated embryos, including a wavy notochord, impaired cartilage and vascular development, lack of hematopoiesis, and defective neurogenesis. Adding copper—but not any other trace mineral—to the water of molecule-treated embryos rescued the phenotypes (Mendelsohn et al., 2006). Establishing these copper deficiency–induced phenotypes in zebrafish greatly facilitated the discovery of zebrafish with mutations linked to copper metabolism. The role of copper in notochord development was suggested to be related to lysyl oxidase, a cuproenzyme that may be important for maintaining notochord sheath integrity (Csiszar, 2001). Lysyl oxidase was further implicated in notochord development through studies of zebrafish knockdown models, in which lysyl oxidase morphants develop a notochord distortion that is similar to copper-deficient fish (Gansner et al., 2007). It is also notable that adding exogenous copper at an early stage of development rescues all defects, suggesting that copper—and likely other metals as well—can enter the embryo from the surrounding water, presumably via transport through the cell membrane.

Using the copper deficiency–linked zebrafish phenotypes as a screening standard (Mendelsohn et al., 2006), a more recent study examined nearly 3000 small molecules and identified a novel panel of copper inhibitors (Ishizaki et al., 2010). Interestingly, the authors combined the zebrafish phenotype screen with a yeast chemical-genetics screen. The molecules that were identified from the zebrafish screen were used to treat a genome-wide library of mutant yeast strains in order to identify novel genetic pathways involved in copper metabolism. Select copper-related genes identified from the yeast screen were then verified using zebrafish knockdown models (Ishizaki et al., 2010). This flexible and powerful combination of zebrafish and yeast chemical-genetics screening approaches will likely be useful in other studies of diseases with identifiable phenotypes.

#### Atp7a

ATP7A plays important roles in exporting excess cytosolic copper from the cell and in the delivery of copper to cuproenzymes via secretory pathways. In humans, mutations in *ATP7A* cause Menkes syndrome, which has a broad spectrum of clinical disorders that are related to copper deficiency, including progressive neurodegeneration, connective tissue abnormalities, and kinky, colorless hair (Tumer and Moller, 2010). After they identified the copper-deficient phenotypes in zebrafish, the same group performed an *N*-ethyl-*N*-nitrosourea (ENU) mutagenesis screen to search for mutants that mimic the chemically induced copper-deficient phenotypes, particularly the pigment loss and wavy notochord. This screen identified the *calamity* mutant, which has the same set of developmental defects. Positional cloning revealed that *calamity* mutants contain a splice variant of the zebrafish homolog of *atp7a*; this alternatively spliced product causes a frame-shift and introduces a premature stop codon (Mendelsohn et al., 2006). In fish, the expression pattern of *atp7a* correlates with the phenotypic defects, with strong expression in the developing notochord. Moreover, the phenotype of the *calamity* mutants can be rescued by overexpressing human *ATP7A*, suggesting conservation of function (Mendelsohn et al., 2006). In addition, *atp7a* zebrafish morphants have the same hypopigmentation and defective notochord phenotype as *calamity* mutants. Finally, a role for Atp7a in modulating the expression of *sp1* and *sod1* has been suggested based on studies of *atp7a* morphants (Chen et al., 2011).

Although the *mottled* mouse is a well-characterized animal model of Menkes syndrome and has been studied for nearly 40 years (Hunt, 1974; Levinson et al., 1994), thanks to its unique properties, the recently developed zebrafish Menkes model has significantly increased our understanding of the underlying disease mechanism. The rapid and *ex utero* development of zebrafish embryos has greatly facilitated the feasibility of performing experiments during early embryogenesis. Embryonic transplantation assays have revealed that transplanted wild-type cells can develop melanin normally in *calamity* mutants, indicating that Atp7a functions cell-autonomously. Furthermore, a combination of copper-suppressing treatment and *atp7a* morpholino injections revealed that the gene dosage of Atp7a determines the animal's sensitivity to copper deficiency (Mendelsohn et al., 2006). These two novel findings obtained using the zebrafish *atp7a* model suggest new therapeutic strategies that focus on tissue-specific gene replacement for treating patients with Menkes syndrome. The zebrafish Menkes model is also a powerful tool for identifying and screening potential compounds that can restore cuproenzyme function in the Atp7a mutant background using chemical screens.

A rescue assay of two zebrafish *atp7a* mutants was conducted via morpholino injection (Madsen et al., 2008). The same ENU screen described above revealed a second allele of *calamity*, and animals bearing this mutation have a phenotype that is similar to animals with the first allele (Mendelsohn et al., 2006; Madsen et al., 2008). Noticing that both *calamity* mutants cause splicing defects, the researchers attempted to rescue the mutants by overexpressing antisense morpholino oligonucleotides designed to specifically target the splice-site junctions in the two mutations. Remarkably, the morpholino injections fully rescued the copper-deficient defects in the *calamity* mutants and permitted the production of wild-type Atp7a protein in all rescued embryos. Nevertheless, although the rescued morphants had decreased amounts of mutant mRNA, they did not have a significant increase in wild-type mRNA, suggesting the presence of competitive translational regulation (Madsen et al., 2008). This study made available promising therapeutic options for using gene correction therapy to treat patients with Menkes syndrome, although the feasibility of such an approach is currently limited by several factors, including the nature of the mutations and the delivery of morpholinos.

Small-molecule copper suppressors used in combination with ENU mutagenesis screens identified zebrafish mutants that are sensitive to limited copper availability (Madsen and Gitlin, 2008). After treating with suboptimal doses of copper chelators (which has no effect in wild-type embryos), developmental defects (including pigment loss and an undulating notochord) were detected in certain mutants that are phenotypically silent with adequate copper supply (Madsen and Gitlin, 2008). Two separate copper-sensitive mutants were identified: a hypomorphic allele of *calamity* and a novel mutant called *catastrophe* (*cto*). Interestingly, in the hypomorphic *calamity* mutants, homozygous embryos from homozygous mothers have reduced pigmentation; this phenomenon does not occur in homozygous embryos from heterozygous mothers, suggesting that the mutant's copper sensitivity is related to deficient loading of maternal copper into the egg. The *cto* mutation is homozygous lethal, with homozygotes developing small, punctate melanocytes; moreover, these animals lose all pigments when treated with copper chelators. *Cto* mutant have a mutation in Atp6, a vacuolar ATPase that is predicted to play a role in proton translocation. Embryonic transplantation of wild-type melanocytes restores normal melanin levels in *cto* mutants, suggesting that the mutants have an intact copper uptake system. However, a suboptimal dose of Atp7a morpholinos completely blocks the production of melanin in the Atp6 mutants, suggesting a novel relationship between the proton transport and copper secretory pathways (Madsen and Gitlin, 2008). This study revealed the mechanisms that underlie copper metabolism by studying mutants that were identifiable only with suboptimal copper supply, and it illustrates the value of using the zebrafish model system to study gene-nutrient interactions.

#### Ctr1

CTR1 is a high-affinity copper importer that was originally identified in yeast (Dancis et al., 1994). Subsequent studies identified human CTR1 as a potent copper transporter that is located primarily in the plasma membrane (Lee et al., 2002). The role of CTR1 in the absorption of dietary copper was further demonstrated using a tissue-specific *Ctr1*-knockout mouse model (Nose et al., 2006). The zebrafish Ctr1 protein shares approximately 70% identity with the human homolog, particularly among the amino acid residues that are essential for copper transport (Mackenzie et al., 2004). Early in development, *ctrl1* expression is generalized, but then becomes concentrated in the intestine, which is consistent with its role in the uptake of dietary copper. *Ctr1*-knockdown fish are embryonic lethal, with massive cell death throughout the neural system, including the brain and spinal cord (Mackenzie et al., 2004). However, the mechanism by which Ctrl1 transports copper in zebrafish, and the protein's role in embryogenesis, requires further study.

## Zebrafish and the metabolism of other trace elements

### Selenium

Selenium plays essential biological functions in the body, primarily in the form of selenoproteins, which contain selenocysteine amino acid residues. These functions include antioxidant defense, thyroid hormone production, and cancer prevention, The mechanisms by which selenium is absorbed and excreted vary based on its chemical form in foods, and these mechanisms are poorly understood (Mehdi et al., 2013).

Several zebrafish selenoproteins have been identified and have a function that is conserved with human homologs, making fish a powerful model for studying selenium metabolism. The expression patterns of more than 20 selenoprotein genes were analyzed in zebrafish embryos, and all of the examined genes have tissue-specific expression patterns, many of which reflect their known functions in mammals (Thisse et al., 2003). Mutations in the human Selenoprotein N-encoding gene *SEPN1* cause various forms of congenital muscular diseases called *SEPN1*-related myopathies, which are characterized by early-onset hypotonia and weakness (Lescure et al., 2009). During somitogenesis, the zebrafish *sepn1* gene is expressed specifically in the somites and notochord, which are the precursors of skeletal muscle and vertebrae, respectively (Thisse et al., 2003; Deniziak et al., 2007). *Seph1*-knockdown embryos have reduced motility, poorly coordinated movement, and poorly delimited myotome boundaries. At the ultrastructural level, morphants exhibit pathological muscle changes, including defects in sarcomere organization and myofiber attachment, as well as altered myoseptum integrity (Deniziak et al., 2007). Thus, the functions of zebrafish *seph1* and mammalian *SEPN1* are strikingly similar, and studies of the zebrafish *seph1* model have yielded new insights into the pathological changes that occur in human *SEPN1*-related myopathy and may serve as an ideal model for future studies of disease mechanisms and treatments.

On the other hand, the zebrafish selenium metabolism has non-mammalian properties as well. Gene duplication is an interesting genetic phenomenon in zebrafish selenium metabolism. Two distinct genes that encode the selenocysteine tRNA^[Ser]Sec^ have been identified (Xu et al., 1999). Selenocysteine tRNA^[Ser]Sec^, the principal component in selenoprotein biosynthesis, is encoded by a single-copy gene in mammals and many other classes. In zebrafish, the two tRNA^[Ser]Sec^ genes have identical coding sequences, and their flanking regions (several hundred bases in length in both directions) are highly homologous, which likely reflects evolutionary gene duplication in teleosts (Xu et al., 1999). Similarly, multiple selenoprotein genes have been detected in fish (Kryukov and Gladyshev, 2000). Two of the zebrafish homologs of the human selenoproteins SEPT—glutathione peroxidase 1 and glutathione peroxidase 4—each have two genes in the zebrafish genome. In addition, the zebrafish *sepp* gene contains duplicated Sec insertion sequence elements and encodes a protein containing 17 Sec residues, which is the largest number of Sec residues in any known protein (Kryukov and Gladyshev, 2000; Tujebajeva et al., 2000). Finally, a novel family of selenoproteins called Sepu was identified in fish, chicken, and many other non-mammalian species, suggesting the divergent evolutionary distribution of selenoproteins in eukaryotes (Castellano et al., 2004). Thus, although the zebrafish is a convenient and powerful model for studying selenium metabolism, researchers must be aware of key genetic differences between fish and mammals.

### Manganese

Manganese (Mn) is a key trace element associated with bone development, superoxide elimination, and the metabolism of amino acids, lipids and carbohydrates. Biologically, manganese functions primarily as a cofactor of various enzymes, including Mn superoxide dismutase (Mn-SOD), glutamine synthetase, and arginase. Mn is transported through the body by transferrin, macroglobulins, and albumin (Fraga, 2005). However, the mechanism of manganese metabolism in animals is poorly understood. The zebrafish Mn-SOD has been cloned and shares 85% sequence identity with the human homolog, including high conservation of the amino acids located in the Mn-binding sites. The fish Mn-SOD gene is highly expressed during the early cleavage stage and has been suggested to be maternally distributed to the eggs, indicating an essential role in embryonic development (Lin et al., 2009). In addition, a Mn-dependent enzyme, ADP-ribose/CDP-alcohol diphosphatase (ADPRibase-Mn), has been functionally characterized in zebrafish (Rodrigues et al., 2012). ADPRibase-Mn family members may play a role in the immune system in vertebrates, as suggested by their expression patterns in the rat (Canales et al., 2008). Similar to the rodent homolog, the zebrafish ADPRibase-Mn is also Mn-dependent and catalyzes the hydrolysis of ADP-ribose and CDP-alcohol. However, the enzyme's cyclic-ADP-ribose hydrolysis activity, which is robust in rat ADPRibase-Mn, is negligible in the fish homolog, possibly due to the lack of any known cyclic ADPR synthesis pathways in fish (Rodrigues et al., 2012).

### Iodine

Iodine is also a non-metal trace mineral. The primary biological role of iodine is a constituent of thyroid hormones, which are essential regulators of body growth, cell metabolism, and body temperature maintenance. Zebrafish are an important model system for studying thyroid development and function. Unlike humans, zebrafish lack a compact thyroid gland. Nevertheless, zebrafish thyroid tissue expresses genes that are critical for its patterning and development, and these genes are conserved with mammals (Porazzi et al., 2009). In zebrafish, thyroid hormones play a role in regulating the differentiation of the pectoral fins and determining the transition from the larval stage to the juvenile stage (Brown, 1997). Thyroid hormones are also essential for the normal function of several physiological systems in fish, including the cardiovascular system, the skeletomuscular system, and the digestive system (Power et al., 2001). Therefore, zebrafish can be an ideal model for studying thyroid diseases and iodine metabolism.

## Conclusions

Due to the availability of powerful genetic tools and its developmental advantages, the zebrafish has become an invaluable model system for studying mineral metabolism. The generation of various zebrafish knockdown and knockout models has greatly facilitated the identification of novel genes and mechanisms that underlie mineral metabolism, particularly with respect to iron and copper, which produce characteristic phenotypic changes when their concentrations are altered. Moreover, the flexible combination of mutagenesis screening and metal chelation treatments, for example in studies of copper metabolism (Mendelsohn et al., 2006; Madsen and Gitlin, 2008), may be useful for identifying representative deficiencies that are related to the metabolism of other metals. In addition, because the zebrafish is an aquatic organism that develops *ex utero*, the endogenous mineral levels in zebrafish can be readily altered by changing the concentration of nutrients in the surrounding water, even as early as embryonic day 0. Thus, the effect of dynamically changing trace mineral levels on early development can be studied quite conveniently in zebrafish (Ho et al., 2012). This advantage also makes zebrafish an ideal tool for studying genetic-nutrient interactions (Madsen and Gitlin, 2008). Furthermore, the zebrafish is a vertebrate organism that is highly conserved with humans, but is small in size and has rapid development, making the zebrafish an ideal tool for studying the functional consequences of gene mutations that have been identified in mammals (De Domenico et al., 2007), for expanding cell-based findings to the systemic/organism level (Devireddy et al., 2010), and for confirming findings obtained from studying lower organisms (Ishizaki et al., 2010). Finally, the results obtained using zebrafish disease models provide important directions for treating human patients (Madsen et al., 2008).

The recent breakthroughs in reverse gene-editing technologies—such as transcription activator-like effector nucleases (TALENs) and the clustered regularly interspaced short palindromic repeats (CRISPR)/Cas9 system—have greatly facilitated the generation of loss-of-function fish mutants, which in turn have enormously accelerated our ability to examine the biological functions of many genes that had a previously elusive relationship with trace mineral metabolism. Future studies such as the construction of zebrafish mutant libraries of mineral transporter proteins will allow researchers to gain important insights into the field of mineral metabolism.

### Conflict of interest statement

The authors declare that the research was conducted in the absence of any commercial or financial relationships that could be construed as a potential conflict of interest.
